# Supplementary material for: Exposure to substance and current substance among school-going adolescents in Timor-Leste
Source: PLOS Glob Public Health. 2022 Aug 4;2(8):e0000797. doi: 10.1371/journal.pgph.0000797 (PMC10021776; doi:10.1371/journal.pgph.0000797)
Supplement: Attachment — Submitted filename: Response to Reviewers.docx [file pgph.0000797.s001.docx]

Dear Editor

Thank you very much for your continuous interest in our study titled, **Exposure to substance and current substance use of school-going adolescents in Timor-Leste: Evidence from the 2015 Timor-Leste Global school-based student health survey**

The authors acknowledge that they revised the manuscript in response to the journal's requirements as well as the editor's and reviewers' comments, and that the necessary changes are listed below.

| **Journal Requirements** | | |
| --- | --- | --- |
| 1. | Please provide additional details regarding participant consent. If you are reporting a retrospective study of medical records, archived samples or third party data, please ensure that you have discussed whether all data were fully anonymized before you accessed them and/or whether the IRB or ethics committee waived the requirement for informed consent. If patients provided informed written consent to have data from their medical records used in research, please include this information | Thank you for your suggestion. This information has been added to the work. You can find them at page 8 subheading “Ethics and data accessibility. |
| 2. | Please review your reference list to ensure that it is complete and correct. If you have cited papers that have been retracted, please include the rationale for doing so in the manuscript text, or remove these references and replace them with relevant current references. Any changes to the reference list should be mentioned in the rebuttal letter that accompanies your revised manuscript. If you need to cite a retracted article, indicate the article’s retracted status in the References list and also include a citation and full reference for the retraction notice. | The references below have added to the reference list  Ajzen I. Theory of planned behaviour. Organ Behav Hum Decis Process. 1991;50:179–211  Jeihooni AK, Sorbi A, Harsini PA, Taghinezhad A, Yari A. The application of theory of planned behavior in improving substance abuse prevention behaviors in male high school students. Curr Psychol. 2021  Balfour J. The role of mesoaccumbens dopamine in nicotine dependence. Neuropharmacol Nicotine Depend. 2015;24:55–98 |
| **Editor** | | |
| 3. | In this study, Bandura’s Social Cognitive Theory has been appropriately selected to provide possible elucidation of the dynamics underpinning the problem phenomenon under study. However, is it not possible that their may be other personal-level dispositions among the population of interest in the study that may play significant role to confirm the researchers’ hypothesis? Additional theoretical considerations may require the inclusion of behavioural control and intention offered by the Theory of Planned Behaviour to confirm or refute the study theoretical position. As the reviewer 1 observed “Alcohol drinking, smoking cigarettes and tobacco, and marijuana are all illegal substances for adolescents, but there are other influencing factors on drinking, smoking, and marijuana use”. I tend to agree on this point considering that the strength of this study is theoretically grounded in the statement of 5th paragraph of the introduction; “We situated our hypothesis within Albert Bandura’s social learning theory (23). The theorist hypothesised that there are both models and learners in every social learning environment. In this social learning context, learners intentionally or unconsciously observe and imitate behaviours from their models” but weakly operationalized in the data collected. The theory has three constructs; observational learning (which was operationalized), self-efficacy expectation and outcome expectancy which were absent in the study tended to weaken it | Thank you for your suggestion. This has been rectified and can be found at page 4-5. We have also updated the limitation to include our inability to analyze essential components of the theory. See this on page 22, line 360-363. |
| **Reviewer 1** | | |
| 4. | In this study, the outcome variable was created by combining four single-item questions measuring students' current use of specific substances including cigarettes, marijuana, alcohol and tobacco.  Alcohol drinking, smoking cigarettes and tobacco, and marijuana are all illegal substances for adolescents, but there are other influencing factors on drinking, smoking, and marijuana use. Please explain why alcohol drinking, smoking of tobacco and cigarettes, and marijuana use were grouped together as an outcome variable. | We agree with you: these illegal substances may have varying influencing factors. However, there are other evidence suggesting similar influencing factors across these different illegal substances, especially amongst adolescents. Additionally, our strategy to combine the individual questions to create our outcome variable was informed by a previous study. This has been explained and can be found at page 4, *“Combination of the four single-item into one variable was based on a similar approach by Moilanen et al. (32)”* |
| 5. | In this study, the terminologies are not unified, for instance, 'child,' 'children,' 'child substance use,' 'child current substance use,' 'adolescent substance use,' etc...  Since this study is targeting adolescents, the terminology, 'child' or 'children' (eg, 'child substance use') needs to be unified with 'adolescent' (eg, 'adolescent current substance use') throughout the whole document. | Thank you for your input. This has been addressed. |
| 6. | In Table 2, please check out the Chi-square value and p-value again (probably, one of the p-value or Chi-square-value is wrong). | This correction has been made. Kindly find this on page 13-14, table 2. |
| 7. | Right under 1.3.3 Multivariable logistic regression, there is typed Table 2 (it will be Table 3). | This has been addressed and can be found on page 14 |
| **Reviewer 2** | | |
| 8. | The study investigated the relationship between exposure to substance and its current usage among school-going adolescents in Timor-Leste country utilizing secondary data from a school-based student health survey conducted in 2015.  Adults and parents were considered as the models, the in-school, adolescents were considered as the learners, while use of the substances was considered as the behaviour.  The authors found that parental tobacco-use and people smoking in the presence of adolescent or second-hand smoking exposure, increased the chance of substance-use among adolescents. Furthermore, being males, adolescents in the upper secondary, and those with food insecurity were more likely to be current substance users whilst adolescents with at least 3 friends and those who experienced parental supervision were less likely to be current users. Overall the study’s concept is brilliant, and apt for the times, the analysis is thorough and the conclusion are appropriately drawn and reflect the analysis performed. However, the study set out to find information that can guide current practice or strengthen policy direction regarding adolescents’ exposure to substance and it current use in Timor-Leste in the year 2021 and going forward. The information these tasks will rely on for its performance were gathered more than 5 years ago. An original study may well be most appropriate for this type of research question. | We are grateful for your review and feedback. |
| 9. | As said in the summary, the data- 2015 utilized for information that is expected to guide or strengthen a current (2021) practice seem relatively old thus making it a significant limitation. This is because behaviour is quite dynamic. Perhaps the scenario should also reflect as a limitation in the study | Thank you for your feedback. This has been added as one of the limitations of the study and you can find it at page 22. *“Although the World Health Organization's current dataset is the 2015 Timor-Leste Global school-based student health survey, future researchers should make an effort to use current datasets done by other agencies because behaviour changes over time and the 2015 dataset does not entirely reflect current behavioural trends.”* |
| 10. | The manuscript at this stage could benefit from line numbering as such a practice makes referencing of queries to become much easier. | Thank you for your feedback. This has been addressed. |
| 11. | One other advantage of using original data for this study would be ability freely select control-variables. For example: presence of cigarettes, marijuana, alcohol and tobacco sales’ shops near schools or homes including being sent on errands to buy these substances. This is a comment for future studies in this direction. | Thank you for your input. These covariates have been recommended for future studies in the write up. |
